# PredPPCrys: Accurate Prediction of Sequence Cloning, Protein Production, Purification and Crystallization Propensity from Protein Sequences Using Multi-Step Heterogeneous Feature Fusion and Selection

**DOI:** 10.1371/journal.pone.0105902

**Published:** 2014-08-22

**Authors:** Huilin Wang, Mingjun Wang, Hao Tan, Yuan Li, Ziding Zhang, Jiangning Song

**Affiliations:** 1 National Engineering Laboratory for Industrial Enzymes and Key Laboratory of Systems Microbial Biotechnology, Tianjin Institute of Industrial Biotechnology, Chinese Academy of Sciences, Tianjin, China; 2 Department of Biochemistry and Molecular Biology, Faculty of Medicine, Monash University, Melbourne, Victoria, Australia; 3 State Key Laboratory of Agrobiotechnology, College of Biological Sciences, China Agricultural University, Beijing, China; 4 ARC Centre of Excellence in Structural and Functional Microbial Genomics, Monash University, Melbourne, Victoria, Australia; University of Alberta, Canada

## Abstract

X-ray crystallography is the primary approach to solve the three-dimensional structure of a protein. However, a major bottleneck of this method is the failure of multi-step experimental procedures to yield diffraction-quality crystals, including sequence cloning, protein material production, purification, crystallization and ultimately, structural determination. Accordingly, prediction of the propensity of a protein to successfully undergo these experimental procedures based on the protein sequence may help narrow down laborious experimental efforts and facilitate target selection. A number of bioinformatics methods based on protein sequence information have been developed for this purpose. However, our knowledge on the important determinants of propensity for a protein sequence to produce high diffraction-quality crystals remains largely incomplete. In practice, most of the existing methods display poorer performance when evaluated on larger and updated datasets. To address this problem, we constructed an up-to-date dataset as the benchmark, and subsequently developed a new approach termed ‘PredPPCrys’ using the support vector machine (SVM). Using a comprehensive set of multifaceted sequence-derived features in combination with a novel multi-step feature selection strategy, we identified and characterized the relative importance and contribution of each feature type to the prediction performance of five individual experimental steps required for successful crystallization. The resulting optimal candidate features were used as inputs to build the first-level SVM predictor (PredPPCrys I). Next, prediction outputs of PredPPCrys I were used as the input to build second-level SVM classifiers (PredPPCrys II), which led to significantly enhanced prediction performance. Benchmarking experiments indicated that our PredPPCrys method outperforms most existing procedures on both up-to-date and previous datasets. In addition, the predicted crystallization targets of currently non-crystallizable proteins were provided as compendium data, which are anticipated to facilitate target selection and design for the worldwide structural genomics consortium. PredPPCrys is freely available at http://www.structbioinfor.org/PredPPCrys.

## Introduction

Solving the three-dimensional (3D) structure of a protein represents a prerequisite and critical step towards complete understanding of its biological function. In addition, knowledge of the 3D structure is useful for research areas that rely on protein structure, such as rational protein design, bioinformatics, biodiversity, and studies on mechanisms of human health and disease [1]. As of July 2013, more than 32 million protein sequences were documented in the NCBI Reference Sequence (RefSeq) database [2]. However, by August 12, 2013, the structures of only 82,146 proteins in the Protein Data Bank (PDB) [3] had been successfully solved using the primary method, X-ray crystallography, accounting for 88.3% of all proteins in PDB. The rapidly increasing sequence-structure gap has resulted in a huge number of structurally uncharacterized proteins. To address this issue, structural genomics (SG), an international initiative, has been applied with the aim of solving the structures of representative members for each of the biologically important protein families [1].

The experimental progress and status of most target proteins in the SG consortium have been made freely available for acceleration of target selection [4]. For example, TargetTrack (http://www.sbkb.org/tt/) is a target registration database that collects information on the experimental progress and status of the selected targets for structural determination by the Protein Structure Initiative (PSI) and other worldwide structural biology projects. TargetTrack combines the TargetDB [5] and PepcDB databases [6], the most widely used records, to extract information in order to develop computational methods for protein solubility and crystallization propensity prediction [7]. As a centralized target database, TargetDB collects protein target data from nine NIH Protein Structure Initiative (PSI) centers and 10 international structural genomics sites [5]. PepcDB (Protein Expression Purification and Crystallization Database) serves as an extension of TargetDB, and provides more detailed historical status and experimental details for each trial [6]. Further descriptions and trial explanations are also available in TargetDB. In addition, other complementary web-based platforms for annotating and exploring targets, such as TOPSAN [8], PSI SGKB [9] and SPINE [10], have been established through the efforts of SG.

As a result of the SG efforts, an increasing number of previously unknown proteins have been structurally solved using X-ray crystallography, NMR spectroscopy and electron microscopy [11]. However, despite the significant progress, only a small proportion of the SG targets have successfully produced high-diffraction quality crystals. For example, as of January 2012, in the SPINE database [10], only about 71.5%, 42.1%, 20.5% and 4.05% of the initially cloned proteins were expressed, solubilized, purified, and successfully produced diffraction-quality crystals, respectively. Failure in the progress of crystallization trials is the major challenge frequently encountered by the SG consortia in structural determination, with setbacks stemming from problems in one or more of the five major experimental steps of cloning, expression, solubility, purification and crystallization. To solve these problems, repeated trial-and-error experiments in a high-throughput mode are commonly performed, which represents a time-consuming and high-cost process [12]. Elucidation of the fundamental principles and biological properties of proteins that govern crystallization should assist in the development of a suitable experimental setup, protocol optimization, and design of improved methods to enhance the success rates of high-quality crystal production [13].

In view of the increasing detailed annotations with respect to both successful and failed attempts to produce high-quality diffraction crystals that can be solved using X-ray crystallography, a variety of analytical, statistical and computational methods have been developed to predict the propensity of each of the five major experimental steps required for crystallization and structural determination. A number of studies have focused on characterization of the important factors influencing the crystallization propensity of proteins. For example, using decision trees and random forests, Goh *et al*. [14] showed that the sequence conservation score across other organisms, percentage of charged residues, occurrence of hydrophobic patches, number of binding partners and sequence length are the most significant factors that influence a protein’s amenability to high-throughput structural determination. Price and colleagues argued that the prevalence of low entropy, well-ordered surface features is the principal determinant of protein crystallization [15]. In summary, large-scale studies to date suggest that prediction of the crystallization propensity of a protein from its sequence is feasible. Nevertheless, a major shortcoming of these methods is that they are mostly developed as simplified predictive models and seldom available as bioinformatics webservers or tools for the wider research community.

Other statistical or machine learning-based crystallization propensity predictors typically use sequence-derived features that can be readily exploited by experimental biologists. Amongst these, SECRET [16] and CRYSTALP [17] predict the crystallization propensity of protein targets with sequence lengths ranging from 46 to 200 amino acids. Additional methods, such as OB-Score [18], ParCrys [19], CRYSTALP2 [20] and MCSG-Z score [21], utilize several types of sequence-derived features to train their models and achieve reasonable computational efficiency and prediction performance. SCMCRYS [22], as a simple voting method, was developed based on the P-collocated amino acid pairs. To further improve performance, some methods, including XtalPred [23], Pxs [15], SVMCRYS [24], PPCPred [13], XANNPred [25], RFCRYS [26] and CRYSpred [27], have incorporated other informative features, such as predicted secondary structure, disorder and solvent accessibility. More recently, Jahandideh *et al*. [28] developed an updated version of XtalPred, namely XtalPred-RF, which used random forest (RF) to train the classifiers based on an enlarged balanced dataset. The results show that RF-based classifiers outperformed those built using support vector machines (SVMs) and artificial neural networks (ANNs). With the rapidly accumulating experimental data generated by SG centers and consequent improvements in protein crystallization technologies, a target previously regarded as non-crystallizable may become crystallizable. Therefore, it is likely that outdated data include some errors in terms of annotation and classification of positive (crystallizable) and negative (non-crystallizable) samples. Indeed, these drawbacks have resulted in performance deterioration when applying previous methods to recently updated data [13]. To address this issue, Mizianty and Kurgan recently developed a new tool designated PPCPred [13] to predict the success of the entire crystallization process, and more importantly, the likelihood of success at each step, using a large updated dataset and comprehensive set of sequence-derived features. Their research revealed important factors that influence success/failure across all the considered steps (e.g., hydrophobicity/hydrophilicity-based indices) as well as individual steps (such as Cys residues for material production and diffraction-quality crystallization, buried His residues for crystallization). However, the PPCPred method suffers from certain limitations. Firstly, despite accuracy on the original dataset, the performance of PPCPred declined substantially when applied to a larger up-to-date benchmark dataset, achieving AUCs (area under the ROC curve) of only 0.683, 0.612, 0.432 and 0.704 for predicting the propensity of protein material failure (MF), purification failure (PF), crystallization failure (CF), and diffraction-quality crystallization (CRY), respectively (shown below). Secondly, given the rapidly accumulating experimental data due to technological advances, there is a pressing need to characterize the critical protein properties that contribute to attempt success at individual steps, and accordingly, develop improved tools to facilitate the high-throughput structural biology efforts of the community.

In the current study, we developed a new sequence-based approach to improve performance and reliability that not only allows prediction of the propensity of the entire crystallization process but also dissects the key features responsible for success at each individual step in protein crystallization and structural determination. This approach, designated PredPPCrys (Prediction of Procedure Propensity for protein Crystallization), combines a wide range of sequence-derived features, including amino acid indices, types, compositions, physicochemical properties, predicted structural features, and other complementary characteristics generated by PROFEAT [5], which have been used for the first time for this purpose to our knowledge. More specifically, PredPPCrys employs a multi-step feature selection and model training procedure based on SVM to eliminate redundant and irrelevant features and highlight the most important factors responsible for failure at each step through the construction of two-level classifiers (first-level and second-level classifiers termed PredPPCrys I and II, respectively). Benchmarking on an enlarged up-to-date dataset extracted from PepcDB, we showed that PredPPCrys outperforms the state-of-the-art predictor, PPCPred, and other existing methods on independent test datasets. The predicted targets of currently non-crystallizable proteins assigned at five difficulty levels (optimal, suboptimal, average, difficult, and very difficult) have been made available at the website, http://www.structbioinfor.org/PredPPCrys/Dataset.html, along with those predicted to pass the five consecutive experimental steps of the crystallization process. We anticipate that the availability of the PredPPCrys web server and classified targets with different confidence scores can be applied to facilitate community-wide efforts for SG target selection.

## Materials and Methods

### Construction of 5-class experimental progress datasets

The PepcDB database provides annotations on experimental progress of protein targets, including status history, reusable text protocols and stop conditions from PSI and other structural biology centers [6]. We downloaded the most recent datasets from the PepcDB database comprising 108,933 targets and 979,645 experimental trials. Each target is defined as the objective of the crystallization trial(s), with each trial representing a set of experimental procedures used to crystallize the target [13]. Our dataset was extracted and selected according to the following criteria. 1) We only selected targets with either a complete stop status ‘current status: work stopped’ or status ‘in PDB’ or ‘crystal structure’, suggesting authentic status of crystallization. The X-ray crystallography-based experimental statuses in PepcDB mainly include ‘selected’, ‘cloned’, ‘expressed’, ‘soluble’, ‘purified’, ‘crystallized’, ‘diffraction’, ‘crystal structure’ and ‘in PDB’. 2) We removed all the trials before January 1, 2006, and after December 31, 2010. Older data were removed to take into account the latest advances in crystallization trials, while new data were removed in cases of incomplete findings and work still in progress. Thus, the annotations regarding experimental status have not been appropriately updated in the database. 3) Trials performed using X-ray crystallography were specifically selected. 4) The most recent and advanced experimental statuses were annotated and used for each target. For example, multiple trials with different statuses may exist for each target, one marked ‘expressed’ as the final status, and more recently, ‘soluble’ as the final status. In this case, we only applied the most advanced status of ‘soluble’ as final and removed the preceding trials. We additionally selected the latest experimental trials for the target among those annotated with the same stop status.

The following 5-class assignments were employed to indicate the experimental failure/success status of crystallization progress for the included targets (Table S1): (1) protein cloning failure (CLF), with the final status annotated as ‘selected’; (2) production of protein material failure (MF), with the final status annotated as ‘cloned’ or ‘expressed’; (3) purification failure (PF), with the final status of ‘soluble’, ‘purified’ or ‘purification failed’; (4) crystallization failure (CF), with the final status of ‘crystallization failed’ or ‘poor diffraction’; and (5) crystallizable (CRYS), with the final status of ‘crystal structure’, ‘structure successful’, ‘crystal structure’ or ‘in PDB’. In particular, the CLF class was used for the first time in this study, while the remaining 4-class system was the same as that applied previously by Mizianty and Kurgan [13]. The major difference between our classification and the earlier 4-class system is that we further distinguished ‘Cloning Failed’ from ‘Production of protein material Failed’. Protein cloning is the crucial first step of protein crystallization, and many proteins fail to pass this step. Therefore, there is a need to discriminate target proteins that fall in this category from those in the ‘Production of protein material Failed’ group. Better understanding of the key factors that account for failures in these two steps is essential.

For 5-class prediction, a target is considered a positive sample if one or more than one experimental trial has passed a given step. Conversely, a target is considered a negative sample if none of the experimental trials succeed in passing a given step. More specifically, in prediction of the protein cloning propensity, target proteins labeled CLF are considered negative, while proteins labeled MF, PF, CF and CRYS are all regarded as positive samples. In prediction of the protein material production propensity, the negatives include all targets marked with trials labeled CLF and MF, while the positives include proteins labeled PF, CF and CRYS. In prediction of purification propensity, we only consider the targets labeled PF as negative samples, i.e., excluding targets labeled CLF and MF, since certain proteins that fail to be cloned or expressed may be purified. Other targets labeled CF and CRYS are considered positives. In prediction of the propensity of crystallization, targets marked with trials labeled CF are regarded as negative, while those labeled CRYS are taken as positive. In prediction of diffraction-quality crystallization propensity, all targets marked as failed trials are regarded as negative, while those labeled CRYS are positive.

Finally, we reduced sequence homology in the datasets by removing sequences with ≥40% sequence identity using CD-HIT [29] within each class. We did not perform this procedure between different classes in order to retain more useful data, as suggested previously [13]. Following this procedure, approximately half the sequences in each class were removed. The final datasets contained 23,348 non-crystallizable and 5,383 crystallizable proteins (See Table S1 for the statistics of the target proteins in each class). To evaluate the performance of our predictors, datasets of the five classes of experimental trials (denoted ‘DB_CLF’, ‘DB_MF’, ‘DB_PF’, ‘DB_CF’ and ‘DB_CRYS’, respectively) were randomly divided into six equally sized subsets, five of which were merged as the benchmark training set (CRYS_train), while the remaining subset was used as the independent test set (CRYS_test). We performed feature selection and parameter optimization of the SVM models via 5-fold cross-validation based on the benchmark training dataset, and evaluated performance with other approaches based on the independent test dataset. In addition, we applied BLAST [30] to further reduce the sequence redundancy between the training and independent test datasets using a cutoff of 25% sequence identity, and assessed the models’ performance on this more stringent independent test dataset. The supplementary file (Supporting Information S1) contains the benchmark training datasets and two different types of independent test datasets.

### Feature extraction

**Figure 1:**
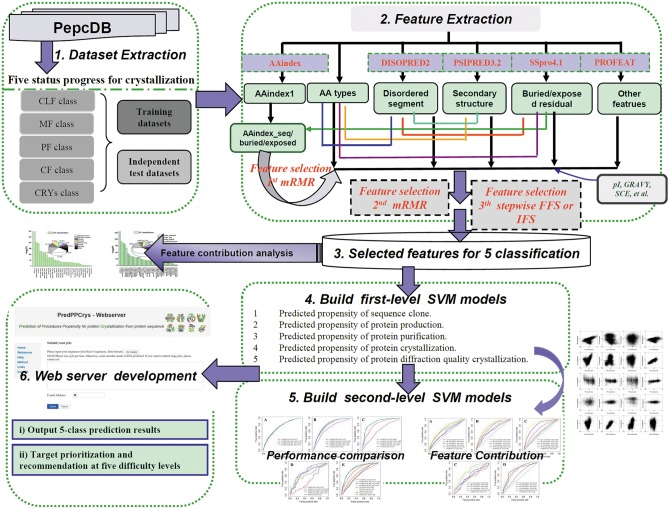
Schematic illustration of the PredPPCrys approach. The details of each of the six major steps are discussed within the main text.

A schematic illustration of PredPPCrys is shown in Figure 1. We extracted a comprehensive set of sequence-derived features as candidate features to train the SVM models of PredPPCrys, with the aim of quantifying the relative importance and contribution of each distinct type of feature or property responsible for the success of each experimental step and overall success of protein crystallization. In total, 2,924 initial features were derived from protein sequences. The complete list of all sequence-derived features is provided in Table S2. A brief summary of the extracted features is provided in subsequent sections.

#### Amino acid types and compositions and physiochemical properties

The compositions of different amino acid types were calculated according to three criteria: (1) composition of 20 standard amino acid types; (2) composition of hydrophobic, hydrophilic, neutral, positively charged and negatively charged amino acids; (3) composition of 10 functional groups according to the amino acid side-chain, such as sulfhydryl (M), phenyl (F/W/Y), carboxyl (D/E), guanidyl (R) (imidazole, primary amino, thiol, amido, hydroxyl and non-polar) [24]. In addition, the dipeptide and tripeptide compositions of the grouped amino acids (rather than the 20 AA types) based on physicochemical properties were calculated (see Table S2 for more details). We additionally used the AAindex database [31] to encode the physicochemical properties of amino acids. The utility of AAindex-based encoding has been confirmed in a number of studies [13], [21], [24], [27], [32]. For example, Creamer [33] showed that side-chain entropy calculated based on the Creamer scale [34], average hydrophobicity value based on the Kyte-Doolittle hydropathy parameters [35] and sequence length are three key factors for protein crystallization, which were also used as features in the current study.

#### Additional complementary features

In addition to the above, we extracted other complementary features [36], [37] using several bioinformatics tools. These included isoelectric point (pI) using Bioperl [38], predicted disordered region using DISOPRED 2 [39], predicted secondary structure using PSIPRED 3.2 [40], and predicted solvent accessibility (residue exposure or burial status) with SSpro 4.1 [41]. Another important aspect was the incorporation of other informative structural and physicochemical features of proteins (a total of 1080 features) calculated with the PROFEAT web server [42], which were used as inputs, along with other features to build SVM models. PROFEAT features included normalized Moreau-Broto autocorrelation, Moran autocorrelation, Geary autocorrelation, transition, distribution, quasi-sequence order descriptors (QAOD), pseudo-amino acid composition (PAAC), amphiphilic pseudo-amino acid composition (APAAC), total amino acid properties (TAAP), and atomic-level topological descriptors (TAAPs). To our knowledge, this is the first study to incorporate all these features for prediction of protein crystallization propensity.

#### Feature combination

Each amino acid displays a characteristic arrangement at both the sequence and structural levels in the protein microenvironment. For example, a hydrophilic residue D is located within a helical segment, predicted to be solvent-buried and intrinsically disordered. Thus, the physicochemical features of this residue would include hydrophilicity, solvent burial, disorder and location in a helical segment, in addition to its microenvironment among other neighboring amino acids, their composition, order, and physiochemical features. We hypothesized that the properties of a residue in a protein are interdependent. As a result, features that combine different types of characteristics may encode key information influencing the propensity of the target protein to pass the five experimental steps (protein cloning, material production, purification, crystallization and diffraction-quality crystal production). Accordingly, we included combinatorial features to train our 5-class SVM models through encoding strategies (for instance, combinations of AAindex properties of amino acids with their predicted burial/exposure status or amino acid types with their predicted secondary structure or the predicted disorder and secondary structure). A complete list of the combinatorial features and explanations is provided in Table S2.

### Feature selection

The large initial feature set may contain some redundant and noisy features, leading to overfitting and overestimation of the performance of machine learning models. Therefore, it is common practice to perform feature selection to isolate a subset of relevant features for prediction [43], [44], [45]. In the current study, we used the mRMR (minimum-redundancy and maximum-relevance) [46] algorithm to rank the initial features. An attractive advantage of mRMR criterion is that it generates a ranked list of the relevant features for prediction in order of importance. mRMR has been widely applied in feature and gene selection in the areas of bioinformatics and systems biology [47], [48], [49], [50], [51]. Following mRMR feature ranking, we performed a two-stage feature selection to efficiently filter out irrelevant features and select the most relevant ones from the initial set. First-stage feature selection was performed based on a two-step mRMR feature selection, while second-stage feature selection was based on incremental and forward feature selection, which is briefly discussed below.

#### Two-step mRMR feature selection

We employed one-step and two-step mRMR strategies to gradually select the relevant features for prediction (Figure 1). For the one-step process, the top 300 contributory and minimum-redundancy features were selected from a total of 2,924 features using the mRMR criterion. For two-step mRMR feature selection, AAindex-based features (including AAindex_seq, AAindex_buried, and AAindex_exposed) were initially used to select the 100 most contributory features from each AAindex-based feature set for predicting the propensity of each individual experimental status class (ultimately, 300 features were selected from the initial AAindex-based features). We subsequently combined the selected 300 AAindex-based feature set with others (2924−3×544 = 1292) in second-step mRMR (the top 300 features were selected after second-step mRMR feature selection). After this procedure, a selected smaller subset of features was subjected to stepwise feature selection.

#### Incremental feature selection (IFS) and forward feature selection (FFS)

We continued to perform an incremental feature selection (IFS) and forward feature selection (FFS) to establish a compact subset of the best performing features. IFS adds a new feature each time to the set according to the ranked importance of all the mRMR-selected features (more important features are added first), and the performance of the resultant SVM predictor based on these feature sets is evaluated in each round. IFS stops when the AUC of the corresponding SVM predictor reaches the maximal value and the selected features contained in this feature subset are considered optimal. For FFS, each candidate feature in the initial set is added to the FFS-selected feature set to build the SVM classifier in each round of FFS. The performance of the resulting predictor is evaluated in each round, and FFS stops until the highest AUC value is reached. The feature set that achieves a higher AUC score than last round is used as the initial feature set for next round. The feature set leading to the highest AUC score is regarded as the optimum set for the corresponding experimental step. Using two-stage feature selection, optimal feature subsets for each of the five experimental steps were selected and used to train the SVM models of PredPPCrys.

### SVM implementation and parameter optimization

For SVM implementation, we used LIBSVM package 2.82 [52] to train and build 5-class SVM predictors. All three types of available kernels in LIBSVM, specifically, sigmoid (SIG), radial basis function (RBF) and polynomial (POLY), were employed to train the models and evaluate their corresponding performance based on the training datasets. We optimized two parameters (*C* and γ) in these kernels using a grid search function implemented by LIBSVM. The optimal feature subset for each class was used to train the models, and performance evaluated with 5-fold cross-validation and independent tests.

#### Construction of first-level and second-level PredPPCrys models with improved performance

The optimal features selected via the two-stage strategy were used as inputs to initially build SVM classifiers of the first-level predictors, termed PredPPCrys I. Next, prediction outputs by PredPPCrys I predictors were used as inputs to build second-level SVM classifiers, termed PredPPCrys II. This two-level framework significantly enhanced prediction performance, as shown in Results and Discussion.

### Performance evaluation

We used the following measures to quantify the performance of the SVM models:
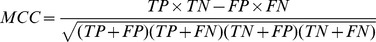


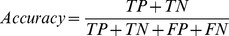


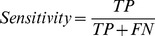


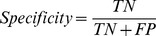


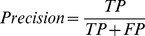
where *TP*, *FP*, *TN* and *FN* are the numbers of true positive, false positive, true negative and false negative, respectively. More specifically, *TP* and *TN* denote the numbers of correctly predicted successful or failed trials of an experimental step, respectively, while *FP* and *FN* signify the number of incorrectly predicted successful or failed trials of an experimental step, respectively. In addition, we used the AUC measure, the area under the receiver-operating characteristic curve (ROC), by plotting the true positive rate (TPR) against the false positive rate (FPR).

AUC is a widely used measure in bioinformatics to evaluate the prediction performance of the trained models especially for imbalanced datasets, which was used as the primary performance measure in this study. In addition, with rapidly accumulated experimental data generated by SG centers and consistently improved protein crystallization technologies, a target previously regarded as non-crystallizable may become crystallizable in the future. Therefore, a real-valued propensity score for a query protein is more important and has more meaning than the classification result of being ‘crystallizable’ or not.

Altogether, the performance of the SVM models was comprehensively evaluated using these six measures based on both 5-fold cross-validation and independent tests. The independent test dataset for the CRYS class was additionally used to evaluate and compare the performance between our system and earlier published methods, since the majority of these methods could not be used to predict the propensity of success in individual experimental steps, with the exception of PPCpred [13]. The other three independent test datasets of MF, PF and CF classes were applied to compare the performance between our method and PPCPred.

## Results and Discussion

### Feature selection results

#### Two-step mRMR feature selection results

Characterization of the important features that determine experimental progress from sequence cloning to acquisition of diffraction-quality crystals that can be structurally solved using X-ray crystallography is critical for understanding the principles that govern protein crystallization. In the current study, we assembled a comprehensive set of sequence-derived features with a total of 2,924 features to conduct an in-depth investigation of the most important factors affecting protein crystallization. We performed two-step feature selection based on mRMR, IFS and FFS strategies to evaluate the relevance and contribution of the features to prediction of target success in steps of the 5-class experimental system.

As mentioned earlier, the initial feature set may contain redundant and irrelevant information. Thus, it is desirable to perform effective feature selection to filter out noisy and redundant features. In this regard, mRMR feature selection has been shown to be a powerful tool for effectively identifying and ranking the most relevant features, with numerous applications over the recent years [47], [48], [49], [51], [53]. The 544 amino acid indices available in the current AAindex1 database [31] represent an abundant information source for the description of physicochemical properties of the 20 amino acids. AA indices are often used as input features in bioinformatics analysis [13], [27], [54], [55]. Nevertheless, some AA indices are highly correlated with each other, exhibiting high correlation coefficients (R) of >0.8. Therefore, to reduce redundancy and irrelevance in AA indices, we performed first-step mRMR feature selection on each AAindex-based feature set. Next, second-step mRMR feature selection was performed to filter out other irrelevant features in the remaining set. The number of selected features after one-step and two-step mRMR methods for 5-class prediction is shown in Table 1.

**Table 1:** Number of selected features after one-step and two-step mRMR feature selection for 5-class prediction.

Feature type	Number of features selected for each class									
	CLF		MF		PF		CF		CRYS	
	A	B	A	B	A	B	A	B	A	B
AAindex1	50	18	110	24	85	31	47	6	110	13
PROFEAT	0	67	22	157	13	147	81	206	23	164
AA composition (AA type 1)	2	5	4	3	2	2	3	1	2	2
AA group (AA type 3)	0	3	2	1	0	0	4	2	2	2
Tri-peptide composition	4	21	12	14	6	11	5	9	8	11
Secondary structure	10	27	25	22	7	13	20	18	25	18
Disorder	6	9	3	5	15	10	2	3	1	7
Exposure related information	105	78	111	43	136	60	76	28	119	52
Burial related information	121	73	7	26	35	24	62	29	5	29
Other	5	4	10	7	5	6	5	3	9	6
Number of some combinationfeatures selected for each class										
AAindex1 & Exposed	95	63	103	34	130	51	68	22	116	44
AAindex1 & Buried	117	60	0	21	31	20	57	25	1	26
AA types & Exposed/Buried	11	23	9	12	6	2	8	7	3	7
Statistical analysis of someselected feature types										
Exposure/Burial ratio	1.15	0.94	0.06	0.60	0.26	0.40	0.82	1.03	23.8	0.56
Percentage ofAAindex relatedfeatures (%)	87.3	47	71	26.3	82	34	57.3	17.7	75.7	27.7

Feature selection was performed based on benchmark datasets.

CLF, MF, PF, CF and CRYS represent assignment of 5-class experimental steps.

A denotes the one-step mRMR feature selection.

B denotes the two-step mRMR feature selection.

AA (amino acid) composition denotes the 20 standard amino acid compositions.

Exposure-related information: the features integrate the predicted exposed residue information.

Burial-related information: the features integrate the predicted exposed residue information.

AAindex 1 & Exposed: average values of physicochemical properties using the amino acid index (AAindex 1) in all the predicted exposed residues (Table S2).

AAindex1 & Buried: average values of physicochemical properties using the amino acid index (AAIndex1) in all the predicted buried residues.

AA types & Exposed/Buried: frequency of the 20 standard AAs (type 1), hydrophobic/hydrophilic/neutral/position/negative AAs (type 2) and AA groups (type 3) in all predicted exposed or buried residues.

Exposure/burial ratio: ratio of the features integrating the predicted exposed residue information to that integrating the predicted buried residue information.

Percentage of AA index-related features denotes the frequency of AA index-related features within the selected set.

Further explanations are included in Table S2.

As presented in Table 1, the proportions of AA index-based features within the 300 selected features in the 5-class prediction system [sequence cloning (CLF), protein material production failure (MF), purification failure (PF), crystallization failure (CF) and crystallizable (CRYS)] were 87.3%, 71.0%, 82.0%, 57.3%, and 75.7%, respectively, after one-step mRMR, and 47.0%, 26.3%, 34.0%, 17.7% and 27.7% (with a decrease in 40.3%, 44.7%, 48.0%, 39.6% and 48.0%, respectively) after two-step mRMR feature selection. This finding indicates that a large proportion of noisy and redundant features contained in the initial AAindex-based feature set is filtered out using the two-step mRMR feature selection. Moreover, this strategy provides more balanced feature selection results for each class, especially with respect to the percentage of exposure-based and burial-based features. For example, for CRYS class prediction, the one-step mRMR method selected 119 exposure-based and 5 burial-based features amongst the top 300 features, while the two-step mRMR method selected 52 exposure-based and 29 burial-based features. The ratio of exposure-based to burial-based features was thus reduced from 23.8 to 0.56. Other features, such as predicted secondary structure, predictor disorder, tri-peptide, PROFEAT features and amino acid compositions were selected for CRYS class prediction after two-step mRMR selection. Therefore, this strategy has the advantage of filtering redundant features and enriching class-specific features. Similarly, the two-step mRMR procedure helps to establish a condensed subset of more useful and relevant features for prediction of the four other classes of experimental steps.

We compared the prediction performance of various SVM models trained using different subsets of features selected with a number of methods (Table 2). Clearly, prediction performance of the SVM models based on feature subsets selected after two-step mRMR achieved higher AUC scores than those based on feature subsets after one-step mRMR across all the experimental steps (including CLF, MF, CF and CRYS), with the exception of the PF class (the underlying reasons for this are unclear). These results suggest that multi-step feature selection is generally useful for reducing feature redundancy and improving the performance of prediction models.

**Table 2:** Performance comparison of the SVM models trained based on various feature subsets selected using different methods on the 5-class benchmark datasets.

Feature selection method	CLF	MF	PF	CF	CRYS
one-step mRMR + IFS	0.691	0.769	0.722	0.684	0.760
one-step mRMR + FFS	0.711	0.767	**0.790**	0.665	0.753
two-step mRMR + IFS	0.698	0.763	0.759	**0.707**	0.756
two-step mRMR + FFS	**0.727**	**0.777**	0.779	0.645	**0.765**

Performance was evaluated based on the AUC score.

#### IFS and FFS feature selection results

On the basis of feature ranking generated with the one-step or two-step mRMR procedure, we subsequently performed IFS and/or FFS [56], [57] to further refine the subsets of selected features for 5-class prediction. As described in Materials and Methods, according to results evaluated using 5-fold cross-validation test for a class, the feature subset based on its corresponding SVM model achieved the highest AUC score was regarded as optimal for this class.

Table 2 depicts the performance comparison of prediction models trained using feature subsets based on one-step and two-step mRMR and IFS or FFS feature selection, in terms of the AUC score. The results suggest that the ‘two-step mRMR + FFS’ feature selection strategy that combines two-step mRMR with FFS criteria outperformed other strategies in CLF, MF and CRYS class predictions, while the best strategy for the PF class was ‘one-step mRMR + FFS’ combining one-step mRMR with FFS criteria. For the CF class, the best feature selection strategy was ‘two-step mRMR + IFS’. The data highlight the importance and necessity of feature selection in the construction of more accurate machine learning models.

After feature selection, a smaller subset of the final selected features for each class was generated for further building the primary SVM classifiers. The prediction performance of the primary classifier evaluated using six measures, specifically, AUC, MCC, Accuracy, Specificity, Sensitivity and Precision, along with the number of final selected features, are presented in Table 3. Overall, 31, 43, 54, 229 and 37 optimal features were finally selected for building the primary classifiers for the sequence cloning (CLF), protein material production failure (MF), purification failure (PF), crystallization failure (CF), and diffraction-quality crystallization (CRYS) classes, respectively. The corresponding AUC scores of the primary classifiers for the five experimental steps were 0.727, 0.777, 0.790, 0.707 and 0.765, respectively.

**Table 3:** Prediction performance of the primary classifier built based on the best-performing final feature subset, along with the number of final selected features for each class.

Class	Numberof final selected features	AUC	MCC	Accuracy (%)	Specificity (%)	Sensitivity (%)	Precision (%)
CLF	31	0.727	0.339	67.8	62.7	71.4	73.3
MF	43	0.777	0.384	70.3	69.6	71.8	50.4
PF	54	0.790	0.445	73.8	70.5	75.5	83.3
CF	229	0.707	0.289	62.7	74.8	58.8	87.8
CRYS	37	0.765	0.309	69.2	69.1	69.3	34.2

Performance on the benchmark training dataset was evaluated based on AUC, MCC, Accuracy, Specificity, Sensitivity and Precision, using 5-fold cross-validation test.

### Prediction performance of first-level PredPPCrys I and second-level PredPPCrys II classifiers

Next, we performed in-depth analysis of the performance of the classifiers based on different kernel functions, with a view to gaining an insight into the important factors that influence the performance of SVM-based models. The training datasets were used to build SVM models of first-level classifiers of PredPPCrys I, which were subsequently tested on the independent test dataset for each class. Three available kernel types, including POLY, RBF and SIG, based on the respective optimal feature subset, were used to build SVM classifiers, and the corresponding parameters (*C* and γ) optimized by grid search. The performance comparison results are shown in Table 4. After parameter optimization, AUC scores of the classifiers improved from 0.717 to 0.728, 0.766 to 0.769, 0.763 to 0.800, 0.681 to 0.701 and 0.750 to 0.770, for the CLF, MF, PF, CF and CRYS classes, respectively. In addition, the best performing classifiers of CLF and PF were built using the RBF kernel, while those of MF, CF, and CRYS were constructed using the POLY kernel.

**Table 4:** Performance comparison of SVM classifiers with different kernel functions and parameters.

Class	Model	POLY			RBF			SIG		
		1/γ	*C*	AUC	1/γ	*C*	AUC	1/γ	*C*	AUC
CLF	Initialmodel	31	1	0.714	31	1	0.717	31	1	0.717
	Optimizedmodel	97	1	0.726	**148**	**1**	**0.728**	183	1	0.727
MF	Initialmodel	43	1	0.766	43	1	0.767	43	1	0.766
	Optimizedmodel	**31**	**1**	**0.769**	38	1	0.768	179	1	0.768
PF	Initialmodel	54	1	0.762	54	1	0.763	54	1	0.761
	Optimizedmodel	2	3	0.795	**1/716**	**0.5**	**0.801**	58	1	0.763
CF	Initialmodel	229	1	0.681	284	1	0.666	284	1	0.654
	Optimizedmodel	**325**	**1**	**0.701**	231	0.2	0.682	252	9	0.693
CRYS	Initialmodel	37	1	0.750	37	1	0.738	37	1	0.750
	Optimizedmodel	**1.34**	**1**	**0.770**	115	0.5	0.754	98	0.125	0.752

Performance was evaluated based on the AUC scores using independent tests.

**Figure 2:**
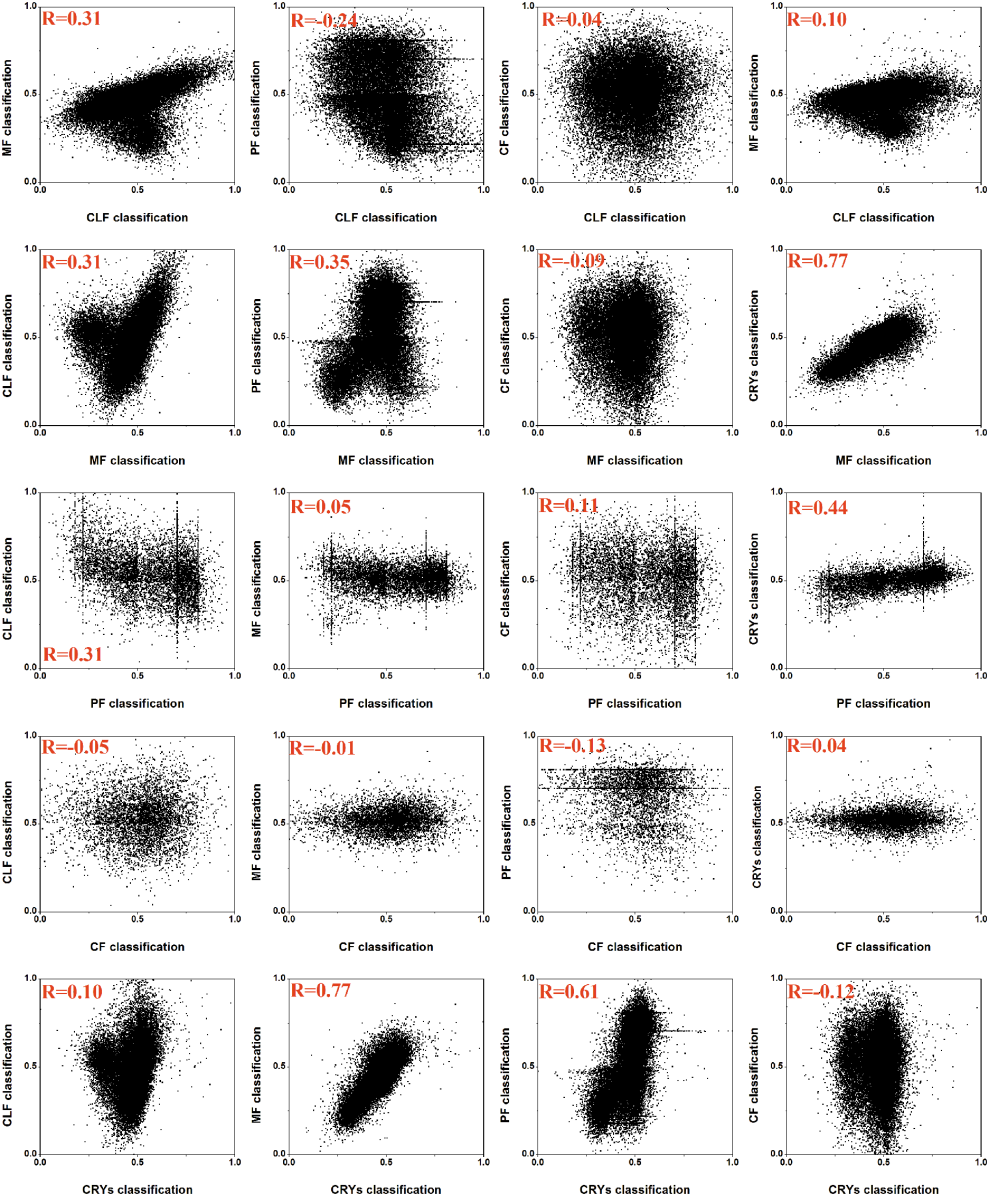
Correlations between the probability outputs of any two classes. Results were evaluated based on the training dataset.

The prediction results generated by PredPPCrys I classifiers with regard to the propensity of success in 5-class experimental steps, from sequence cloning to high-diffraction quality crystal yield, can be used to analyze the inter-dependence or inter-correlation between any two steps in experimental trials. To achieve this, we calculated the correlation coefficients of the probability outputs of the SVM classifiers between any two classes (Figure 2). For example, for CLF class prediction, MF is the most inter-correlated class with a correlation coefficient R of 0.31, while for MF class prediction, CRYS is the most inter-correlated class with a correlation coefficient R of 0.77 (Figure 2).

**Figure 3:**
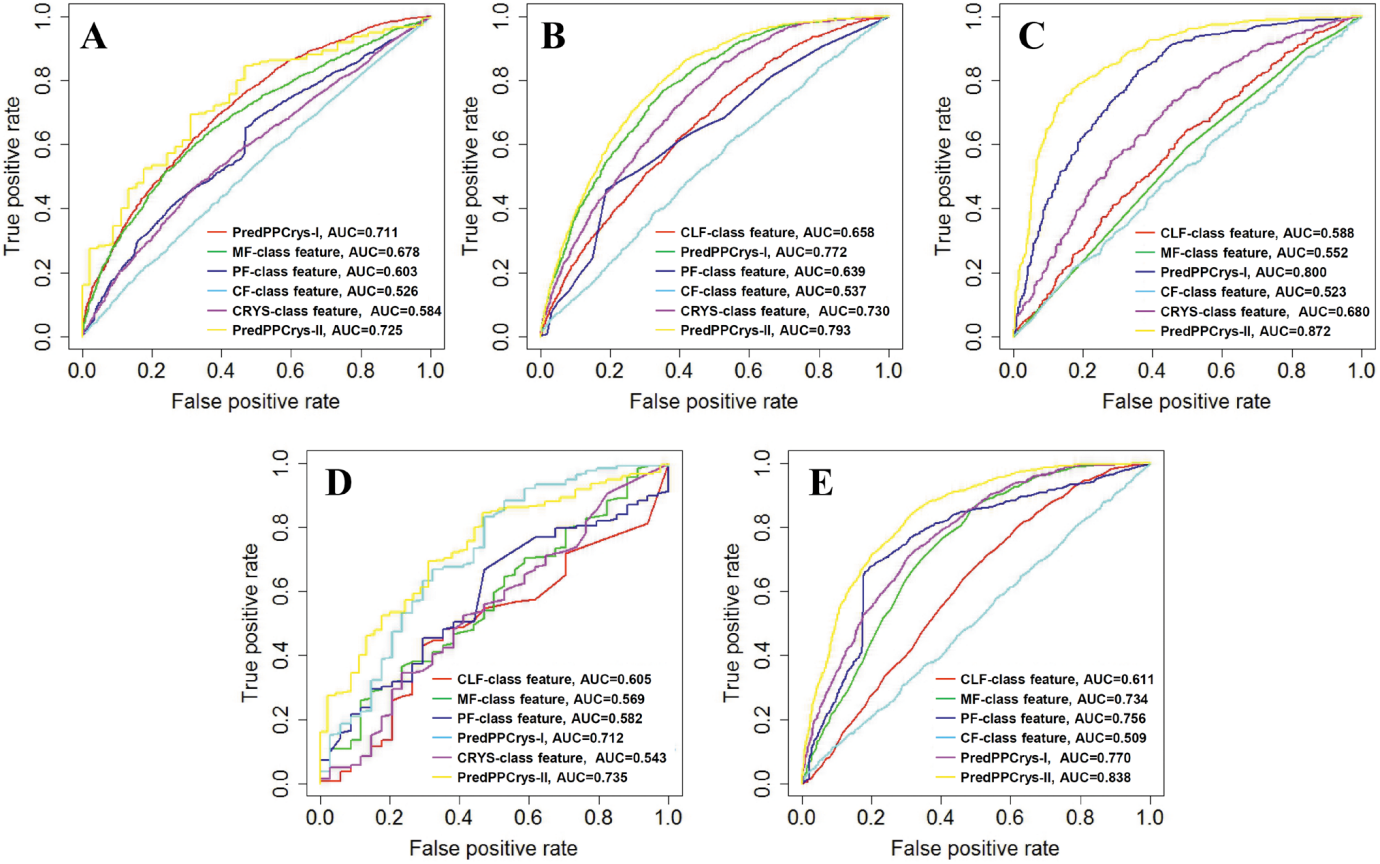
ROC curves for different predictors. (A), CLF; (B), MF; (C), PF; (D), CF; and (E), CRYS class. Taking the CLF class as an example, the performance of the first-level predictor PredPPCrys I (corresponding to the CLF class feature in Figure A), predictors built using the outputs of classifiers for other classes as inputs, as well as the second-level predictor, PredPPCrys II, are compared using the respective ROC curves. All predictors were built using the optimized SVM parameters based on the respective training datasets, and subsequently tested on the corresponding independent test datasets.

To further illustrate this finding, we trained classifiers using the prediction outputs of other classes as the input features and evaluated the performance of the resulting classifiers. ROC curves illustrating classifier performance are presented in Figure 3. Taking the CLF class as an example, the classifier using the output of the MF class as input achieved an AUC score of 0.678 for predicting the CLF class, while the AUC score of the PredPPCrys I predictor was 0.711 (Figure 3A). These results consistently suggest that the outputs of classifiers for other classes are beneficial for further improving the prediction of a given class.

To confirm this, we generated second-level PredPPCrys predictors using the predicted outputs of the first-level PredPPCrys predictors as input features, as described in Materials and Methods. Figure 3 illustrates the prediction performance comparison between the first-level and second-level predictors. The results confirm performance improvement across all five classes, with improved AUC scores from 0.711 to 0.725, 0.772 to 0.793, 0.800 to 0.872, 0.712 to 0.735 and 0.770 to 0.838, respectively. Clearly, PredPPCrys II consistently outperforms PredPPCrys I predictors by exploiting outputs from the first-level predictors as inputs for the second-level predictors.

### Comparison of PredPPCrys with previous methods

As mentioned earlier, some proteins that previously failed in crystallization trials may become crystallizable and produce diffraction-quality crystals with the aid of advanced experimental technologies. This highlights the importance and necessity of constructing updated independent test datasets that reflect the true results of their crystallization status. Here, we constructed new independent test datasets, and compared the prediction performance of our methods (PredPPCrysI and three available optimized kernel models of PredPPCrys II) with other previously published methods, including ParCrys [19], OBScore [18], CRYSTAP2 [20], XtalPred [23], SVMCRYs [24], PPCPred [13], SCMCRYS [22], and XtalPred-RF [28]. The performance of all predictors was evaluated using AUC, MCC, Accuracy, Specificity, Sensitivity and Precision measures based on independent tests, and results are summarized in Table 5. Since most of the other methods (except PPCPred) can only be used to predict crystallization propensity, we mainly compared performance for this particular class (Table 5). A list of the sequence-derived features used by the different methods is presented in Table S3.

**Table 5:** Performance comparison of PredPPCrys I, PredPPCrys II and previous methods, including PPCPred, ParCrys, OBScore, CRYSTAP2, XtalPred, SVMCRYs, SCMCRYS and XtalPred-RF.

Experimental step	Method	AUC	MCC	Accuracy (%)	Specificity (%)	Sensitivity(%)	Precision (%)
CLF	PredPPCrys I	0.711	0.296	65.33	63.58	66.50	73.16
	PredPPCrys I (−)	0.697	0.291	64.70	64.40	64.94	70.48
	**PredPPCrys II**	**0.725**	**0.322**	**66.54**	**65.56**	**67.20**	**74.44**
	PredPPCrys II (−)	0.710	0.307	65.66	64.40	66.61	71.01
MF	PPCPred	0.683	0.334	68.06	67.99	68.22	47.20
	PredPPCrys I	0.772	0.380	69.93	68.21	72.88	49.95
	PredPPCrys I (−)	0.776	0.398	69.86	67.37	75.03	52.47
	**PredPPCrys II**	**0.793**	**0.416**	**71.95**	**71.36**	**73.30**	**52.70**
	PredPPCrys II (−)	0.809	0.461	74.32	74.42	74.10	58.18
PF	PPCPred	0.612	0.183	58.83	62.23	57.08	74.57
	PredPPCrys I	0.800	0.460	74.83	70.52	77.02	83.77
	PredPPCrys I (−)	0.779	0.437	72.85	72.65	72.95	83.89
	**PredPPCrys II**	**0.872**	**0.579**	**79.73**	**81.43**	**78.86**	**89.31**
	PredPPCry II (−)	0.872	0.588	80.22	82.55	79.09	90.31
CF	PPCPred	0.432	−0.014	55.23	32.21	61.24	75.53
	PredPPCrys I	0.712	**0.280**	67.05	67.65	66.91	89.42
	PredPPCrys I (−)	0.693	0.258	66.04	65.63	66.14	88.42
	**PredPPCrys II**	**0.735**	0.175	**69.47**	**68.89**	**69.50**	**97.80**
	PredPPCrys II (−)	0.692	0.186	59.12	65.63	57.48	86.90
CRYS	ParCrys	0.611	0.132	59.66	60.56	55.91	25.40
	OBScore	0.638	0.184	59.28	57.78	65.49	27.14
	CRYSTAP2	0.599	0.123	51.64	48.10	67.78	22.28
	XtalPred	-	0.224	65.04	65.61	62.51	29.31
	SVMCRYs	-	0.142	55.11	52.78	65.70	23.39
	PPCPred	0.704	0.254	63.63	62.09	70.67	29.03
	XtalPred-RF	-	0.205	60.94	59.67	66.41	27.56
	SCMCRYS	-	0.145	60.93	62.01	56.24	25.48
	PredPPCrys I	0.770	0.326	69.65	69.30	71.13	35.23
	PredPPCrys I (−)	0.794	0.379	72.63	73.30	70.32	43.46
	**PredPPCrys II**	**0.838**	**0.428**	**76.04**	**76.21**	**75.30**	**42.64**
	PredPPCrys II (−)	0.858	0.502	78.35	78.16	79.02	51.35

Performance was evaluated based on independent test datasets.

(−) denotes that our proposed method PredPPCrys was tested on the independent test datasets with a 25% sequence identity cutoff compared with the training datasets.

**Figure 4:**
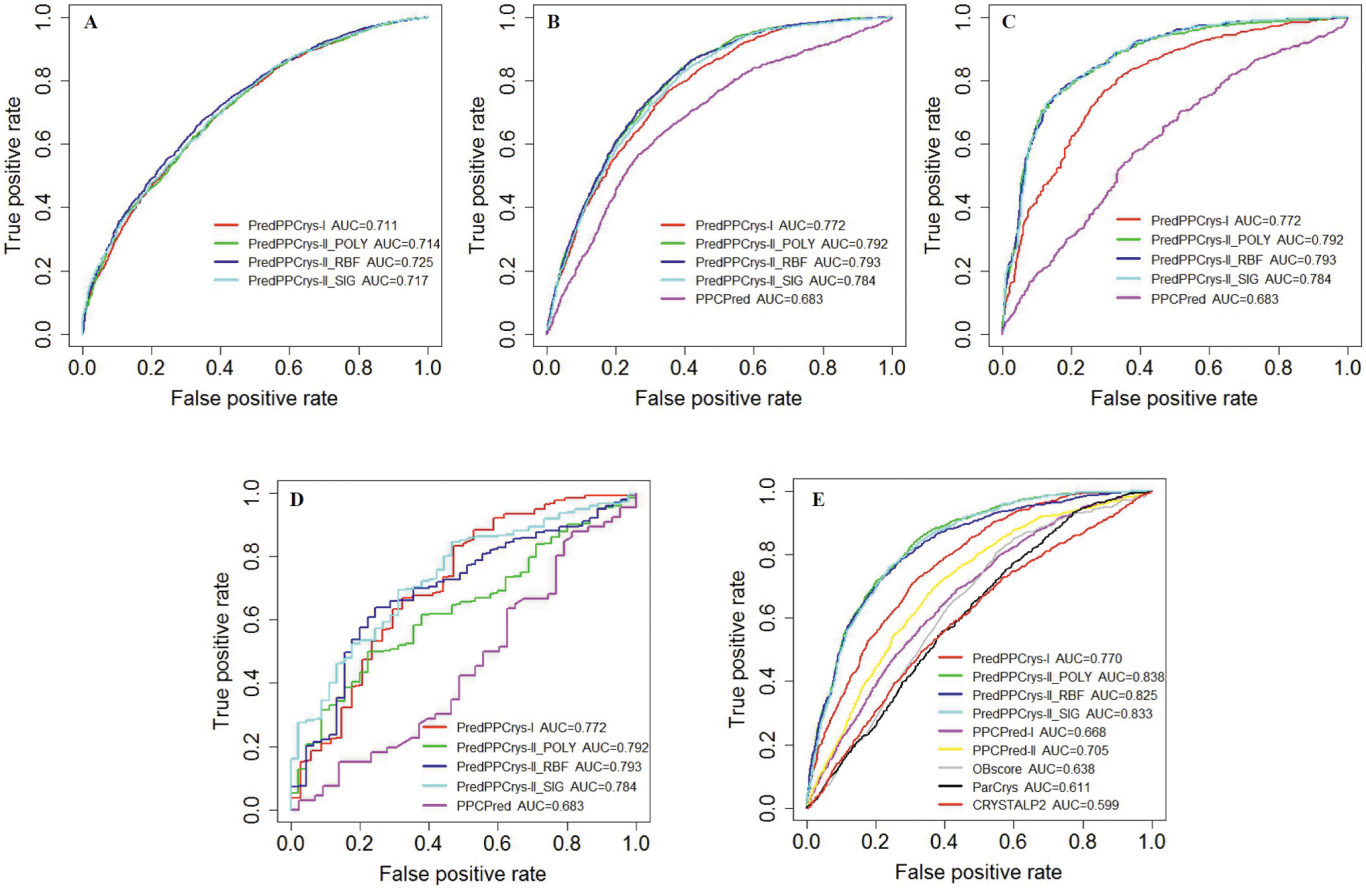
ROC curves displaying the performance of our methods (PredPPCrys I and II predictors), compared to previous procedures, on independent test datasets for predicting propensity of targets to successfully pass each experimental step. (A), CLF; (B), MF; (C), PF; (D), CF and (E), CRYS class. PredPPCrys-I denotes the first-level predictors of PredPPCrys, PredPPCry-II denotes second-level predictors of PredPPCrys, while PredPPCrys-II_POLY, PredPPCrys-II_RBF, PredPPCrys-II_SIG denote the best performing SVM classifiers built with SVM_POLY, SVM_RBF, SVM_SIG kernels in second-level predictors, respectively.

ParCrys, OBScore and CRYSTAP2 achieved AUC scores of 0.611, 0.638, and 0.599, respectively, while XtalPred, SVMCRYs and SCMCRYS achieved MCC values of 0.224, 0.142 and 0.145, respectively, for predicting the propensity to yield diffraction-quality crystals (CRYS). Most recently, XtalPred-RF, an updated version of XtalPred, was developed using a RF algorithm based on a new balanced dataset. It was found to achieve a better performance on the balanced dataset, but was shown to perform worse on the imbalanced dataset. Clearly, the two variants of PredPPCrys (PredPPCrys I and II predictors) and PPCPred significantly outperformed the other methods when evaluated using AUC and MCC scores. Furthermore, PredPPCrys II predictors performed the best among all the methods, followed by PredPPCrys I predictors. PredPPCrys II achieved the highest AUC of 0.838 and MCC of 0.428, which were 19% and 68% increased, compared to the corresponding values of PPCPred. Additionally, PredPPCrys II achieved the highest specificity (76.21%), sensitivity (75.30%) and precision (42.64%) values, relative to the other methods. Analysis of the ROC curves obtained at varying Specificity/Sensitivity values (Figure 4) led to the same conclusions.

Since we introduced 5-class prediction in this study, none of the previous methods could be applied to predict the likelihood of sequence cloning failure (CLF class) for comparison with PredPPCrys. However, we were able to compare the performance of PredPPCrys with the state-of-the-art method, PPCPred, for predicting the propensity of [success in] other experimental steps (MF, PF and CF), in addition to CLF and CRYS. The results are shown in Table 5. PredPPCrys II outperformed PPCPred, with higher AUC (0.793, 0.872, and 0.735 vs. 0.683, 0.612, and 0.432, respectively) for each of the three classes. The ROC curves displayed in Figure 4 clearly indicate that PredPPCrys II compares favorably with PPCPred and PredPPCrys I.

In addition, to assess the influence of sequence similarity between the training dataset and independent test datasets on the prediction performance of PredPPCrys, we further reduced the sequence redundancy between the training and testing datasets using a sequence identity cutoff of 25% and tested the performance of PredPPCry models on the new testing datasets. As shown in Table 5, there was a slight decrease in the performance on the independent test datasets of 40% and 25% sequence identity, as evaluated by AUC and MCC scores. However, this performance difference was not significant. These results indicate that PredPPCrys could achieve a robust performance when being applied to predict query sequences with lower sequence similarity to the training datasets.

As previously described, only a small number of proteins can successfully yield high-diffraction quality crystals (HCDC), while most of them failed in the procedures of protein expression, solubility, purification, and production of diffraction-quality crystals. Therefore, we want to develop PredPPCrys in this study for the purpose of accurately predicting and selecting potential targets with larger likelihood of yielding HDQC from a large number of current non-crystallizable proteins, similar to the previous work of PPCPred. For this purpose, we employed an imbalanced database to train models, which can be employed to prioritize all current non-crystallizable structural genomics targets.

Recent studies have shown that the methods developed using RF classifiers achieve better performance for predicting protein crystallizability. RFCRYS and XtalPred-RF are such methods that performed well particularly when tested on the balanced datasets. Jahandideh *et al* [28] extracted a new larger dataset (e.g. the XtalPred-RF dataset) from the PSI TargetTrack database. This balanced dataset was generated by reducing negative data and had nearly equal counts of negative and positive samples. Therefore, in this study, we also applied our method to the XtalPred-RF dataset and compared the performance between different methods. As a result, PredPPCrys model trained using the optimal features selected by multi-step heterogeneous feature selection achieved an MCC value of 0.478, which was slightly higher than XtalPred-RF (MCC = 0.470) (see Table S4). These results indicate that with the efficient multi-step feature selection, PredPPCrys is able to provide a competitive performance for protein crystallization prediction compared with recently developed predictors.

### Feature contribution to the 5-class prediction system

**Figure 5:**
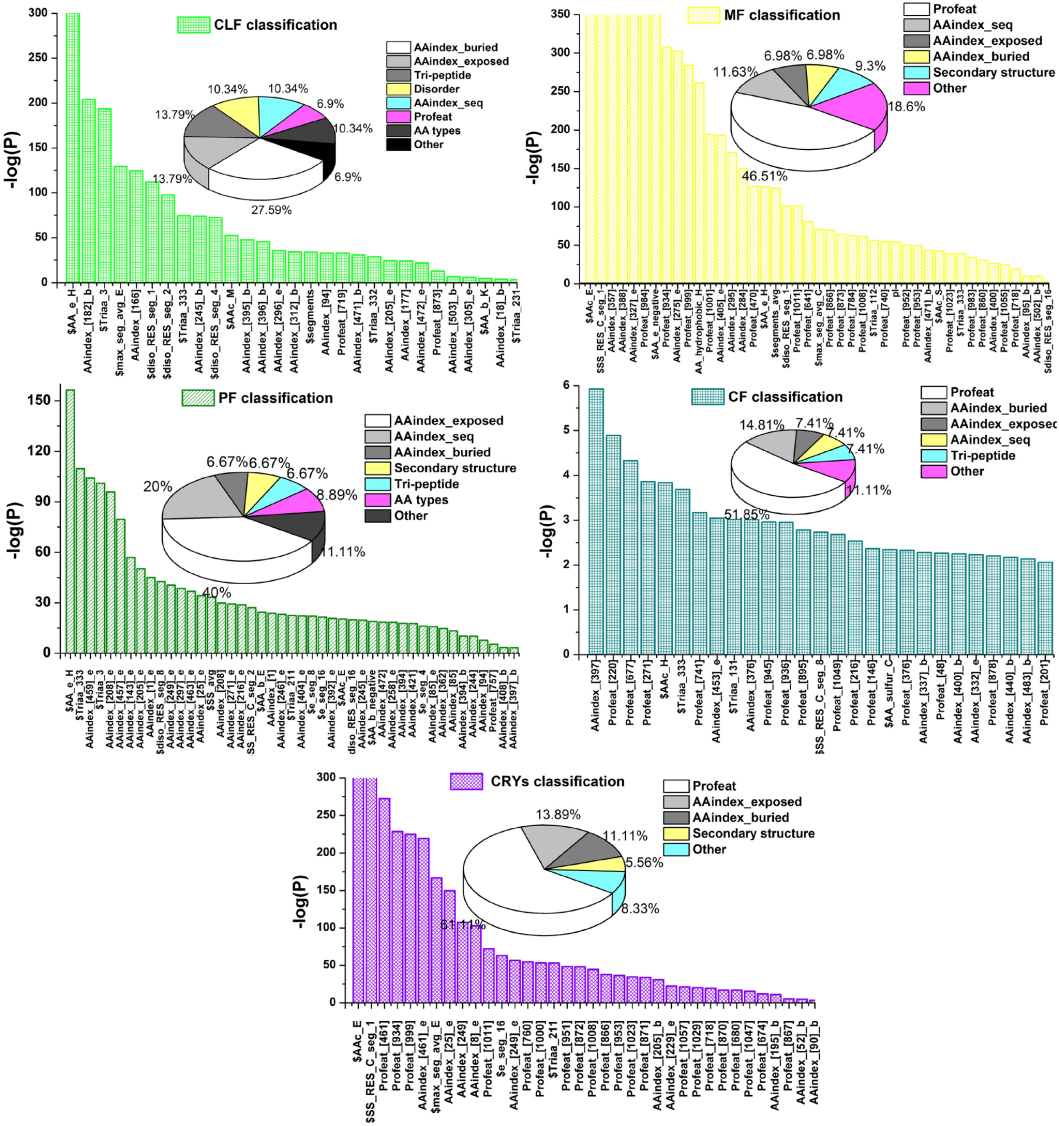
Statistical significance of the contributions of selected features to the prediction performance of the five classes, evaluated based on the negative logarithmic value of *p*-value (-log(*P*)) calculated using *t*-tests. Contribution significance was determined using *t*-tests, and only the final selected feature types that made a significant contribution (*p*<0.01) to performance were included in the analysis. The vertical and horizontal axes display the contributory features. The pie chart insets denote the percentages of selected feature types in the final feature subset for each class.

We further analyzed the contributory effects of the final set of selected features to the prediction performance of PredPPCrys. Features were analyzed in three broad categories, namely, PROFEAT, AAindex and other features, as shown in Fig. 5. For details of the final selected features for the five classes, please refer to the Supplementary files at http://www.structbioinfor.org/PredPPCrys/Datasets.html.

To our knowledge, PROFEAT features have been used for the first time in this study. The initial PROFEAT features included dipeptide composition (Profeat[1–400]); normalized Moreau-Broto autocorrelation (Profeat[401–490]); Moran autocorrelation (Profeat[491–580]); Geary autocorrelation (Profeat[581–670]); composition (Profeat[671–691]), transition (Profeat[692–712]), distribution (Profeat[713–817]) of hydrophobicity, Van der Waals volumes, polarity, polarizability, charge, secondary structure and solvent accessibility; quasi-sequence order descriptors (Profeat[818–977]); amphiphilic pseudo-amino acid composition (Profeat[978–1057]); and total amino acid composition (Profeat[1058–1060]). Each feature was numbered in accordance with that provided by the PROFEAT webserver. Among the features, dipeptide compositions have been previously used for protein crystallization prediction [16], [17], [20], [21], which are especially important for predicting the propensity of the CF class, accounting for 25.93% of the selected features for this class.

Interestingly, a few autocorrelation-based features (Profeat[401–670]) were found to be significant for the MF class, with significant *p*-values, (Profeat[470]) = 1.7×10^−127^ and (Profeat[641]) = 1.2×10^−81^, and *p*-value (Profeat[461]) = 4.7×10^−273^ for the CRYS class. This finding suggests that the features describing protein–protein interface properties play an important role in influencing protein material production and diffraction-quality crystal preparation processes. Moreover, other features of PROFEAT that describe the composition, transition and distribution of 7-type properties (Profeat[671–817]) were relevant for the performance of the predictors of respective classes, including Profeat[719] for CLF, Profeat[784,718] for MF, Profeat[757] for PF, Profeat[677,741] for CF and Profeat[760,680,674] for CRYS. Quasi-sequence order descriptors and amphiphilic pseudo-amino acid compositions appeared to play important roles in the prediction of nearly all classes, accounting for 3.4%, 30.2%, 18.5% and 38.9% of the selected features for CLF, MF, CF and CRYS, respectively.

The second largest feature category, specifically, AAindex-based, was also critical for prediction performance. These features can be further divided into those describing the physicochemical properties of buried residues (denoted as ‘AAindex_buried’), exposed residues (denoted as ‘AAindex_exposed’) and whole protein (denoted ‘AAindex_seq’ in Figure 5). Analysis of these AAindex features revealed several important findings. For example, for the CLF class, the AAindex features that describe physicochemical properties of buried residues were more abundant than those of exposed residues and whole protein (27.59%, compared with 13.79% and 10.34%, respectively). Similarly, for the CF class, the AAindex-based properties of the buried residues were more abundant than exposed residues and whole sequence.

Interestingly, for the PF and CRYS classes, the physicochemical properties of the exposed residues of the protein appeared to play more important roles in predicting propensity. During the processes of protein purification and crystallization, higher concentrations of soluble proteins are often required and the physicochemical properties of the exposed parts of the protein may influence solvent accessibility and protein–protein interactions. From this perspective, the significant role of these properties is understandable. In addition, the AAindex properties of the whole sequence (11.63%) were abundant in the feature subset for the MF class, suggesting that these properties are more useful for predicting protein material production propensity. Subdivision of protein properties into these three subtypes is therefore more informative and aids in improving prediction performance.

Other final selected feature types critical for prediction included predicted disorder, amino acid types, predicted secondary structure, and tripeptide compositions (Table S2). The disorder segment-based features were previously found to be particularly useful for MF, CF and CRYS classes by Mizianty and Kurgan [13]. In the current study, these features were selected in the final feature subsets and were more important, not for MF, CF and CRYS as suggested earlier, but for CLF and PF classes (Figure 5). In particular, the disorder segment-based features led to significant improvements in the prediction performance of CLF and PF classes, compared with the former two classes.

Another important feature type relevant for prediction is the predicted secondary structure. This includes, for example, the coil segment divided by sequence length (denoted ‘$SS_RES_C_seg_1’, with *p*-value≈0) for MF and CRYS classes, length of the maximal β sheet (denoted ‘$max_seg_avg_E’) for CLF class, length of maximum coil segment (‘$max_seg_avg_C’) for MF class, total secondary structure segments (‘$SS_avg’) for PF class, frequency of sulfur containing amino acids in the coil segments (‘$AA_sulfur_C’) and frequency of more than 8 AA coil segments (‘$SS_RES_C_seg_8’) for CF class. These results are in agreement with previous data [13], [23], [24], [58]. In addition, we found that tripeptide compositions based on hydrophobic (1: amino acids coded F/I/M/L/V/M/Y/C/A), hydrophilic (2: amino acids coded R/K/N/D/E/P) and neutral (3: amino acids coded T/H/G/S/Q) types were useful for predicting propensity for CLF, PF, CF and CRYS classes. Tri-peptide compositions were used for the development of CRYSTALP2 [20] and SVMCRYS [24].

Notably, the frequency of exposed His residues additionally made a significant contribution to the prediction of success in CLF, PF and MF classes, with *p*-values of 0, 3.4×10^−157^ and 5.9×10^−127^, respectively. These results are consistent with the findings of Mizianty and Kurgan [13]. Interestingly, the frequency of Glu residues in a protein was identified as an important factor for predicting target propensity for MF and CRYS classes. Furthermore, the frequency of Glu residues buried partly or wholly was a critical factor for PF. Consecutive exposed segment was another important factor for CLF (with more than one exposed segment), PF (segments longer than 4, 8 or 16 AAs) and CRYS (segments longer than 16 AAs) classifiers, indicating that longer exposed segments are favorable for protein purification and diffraction-quality crystal yield. Finally, the isoelectric point (pI) of the protein was critical for prediction of protein material production failure, consistent with previous data [13], [16], [18], [20].

In summary, this study has not only reinforced the importance of several major feature types reported as critical for prediction (e.g., dipeptide compositions, tripeptide compositions, predicted secondary structure and isoelectric point of the protein), but also revealed other complementary features that have not been previously recognized as significant (e.g., autocorrelation-based PROFEAT features). These features were selected using an efficacious and reliable multi-step procedure to obtain good prediction performance of models that were trained and tested on up-to-date experimental datasets. Through multi-step feature selection analysis, we provided in-depth details of the relative contribution and significance of the selected features and generated optimally performing feature subsets for each major experimental step.

We evaluated the contribution of each individual feature by examining the AUC score of the predictor that used only a feature as the input using benchmark datasets. The feature that resulted in a higher AUC score of the corresponding model was more important. The results are shown in Figure S1. We can see that the AUC-based results were generally consistent with that obtained based on *p*-values.

### Implementation of the online web server and prioritization of all non-crystallizable structural genomics targets for PredPPCrys

To date, yielding diffraction-quality crystals of structural genomics targets for structure determination has remained a formidable task for biologists. To facilitate high-throughput SG efforts, we have constructed an online prediction web server of PredPPCrys at http://www.structbioinfor.org/PredPPCrys/server.html, which is programmed using Perl. Usage of the webserver starts with input of sequences in the FASTA format. A typical prediction task will normally take 3–5 min to complete. The prediction output includes the predicted probability scores of the propensity of the query protein to pass each of the five experimental steps. In particular, a protein is categorized into one of the five possible crystallization propensity classes according to the prediction cutoffs: ‘Optimal’ (probability score ≥0.6), ‘Suboptimal’ (0.55≤ probability score <0.6), ‘Average’ (0.45≤ probability score <0.55), ‘Difficult’ (0.2≤ probability score <0.45) and ‘Very difficult’ (probability score <0.2). The probability score output by the web server allows users to rank and prioritize their own targets.

We subsequently applied the best performing PredPPCry predictors to rank and annotate SG targets that have not successfully yielded diffraction-quality crystals. The prediction results of all prioritized targets by PredPPCrys are freely available at the website (http://www.structbioinfor.org/PredPPCrys/Datasets.html). The statistics of prioritized structural genomics targets with different prediction cutoffs are presented in Table S5. Proteins annotated ‘Optimal’, ‘Suboptimal’, ‘Average’, ‘Difficult’ and ‘Very difficult’ accounted for 5.1%, 7.7%, 19.9%, 46.4% and 20.9% of the total targets, respectively. We believe that the availability of computationally prioritized targets will expedite the determination of 3D structures and consequent functional characterization of these proteins.

## Conclusions

In this study, we have developed a new approach, termed PredPPCrys, to predict the propensity of a protein to pass the five critical consecutive experimental stages involved in the crystallization process, including sequence cloning, protein material production, purification, crystallization and diffraction-quality crystal yield. By taking into account the contribution of a large number of heterogeneous features and subsequently applying multi-step feature selection using mRMR, IFS and FFS methods, PredPPCrys achieved better prediction performance, compared with other published methods, on independent test datasets. As one of the only two methods for predicting the propensity of targets to pass each individual experimental step, PredPPCrys also outperformed the state-of-the-art PPCPred method, with an AUC of 0.838, Matthews correlation coefficient of 0.428 and overall accuracy of 76.04% for predicting crystallization propensity. Moreover, the webserver of PredPPCrys has been made publicly available at http://www.structbioinfor.org/PredPPCrys, which is expected to be a powerful tool to facilitate and accelerate ongoing SG efforts.

## Supporting Information

Figure S1 **The importance and contribution of final selected features to the prediction performance of five classes, as evaluated by the AUC score using benchmark datasets.** In particular, the optimal feature set of the CF class included 229 features and accordingly, we only displayed the features with AUC scores of larger than 0.55, in order to show the results more clearly.
(TIF)

Table S1 **Statistics of the 5-class assignment of protein targets marked with the corresponding statuses, including Sequence cloning failed (CF), Production of protein material failed (MF), Purification failed (PF), Crystallization failed (CF) and High-quality diffraction crystallization (CRYS).** The data were extracted from PepcDB in this study.
(DOCX)

Table S2 **A full list of all the considered sequence and sequence-derived features in this study.**
(DOCX)

Table S3 **List of the sequence-derived features used in other previous studies and our study.**
(DOCX)

Table S4 **Performance comparison of binary classification results of crystallizability on the independent test set of the XtalPred-RF method.**
(DOCX)

Table S5 **Summary results of target selection on presently non-crystallization proteins by PredPPCrys.**
(DOCX)

Supporting Information S1 **The file contains the benchmark training datasets and two different types of independent test datasets (with sequence identity cutoffs of 25% and 40%, respectively).**
(ZIP)
