# Supplementary figures and images for: PredPPCrys: Accurate Prediction of Sequence Cloning, Protein Production, Purification and Crystallization Propensity from Protein Sequences Using Multi-Step Heterogeneous Feature Fusion and Selection
Source: PLoS One. 2014 Aug 22;9(8):e105902. doi: 10.1371/journal.pone.0105902 (PMC4141844; doi:10.1371/journal.pone.0105902)

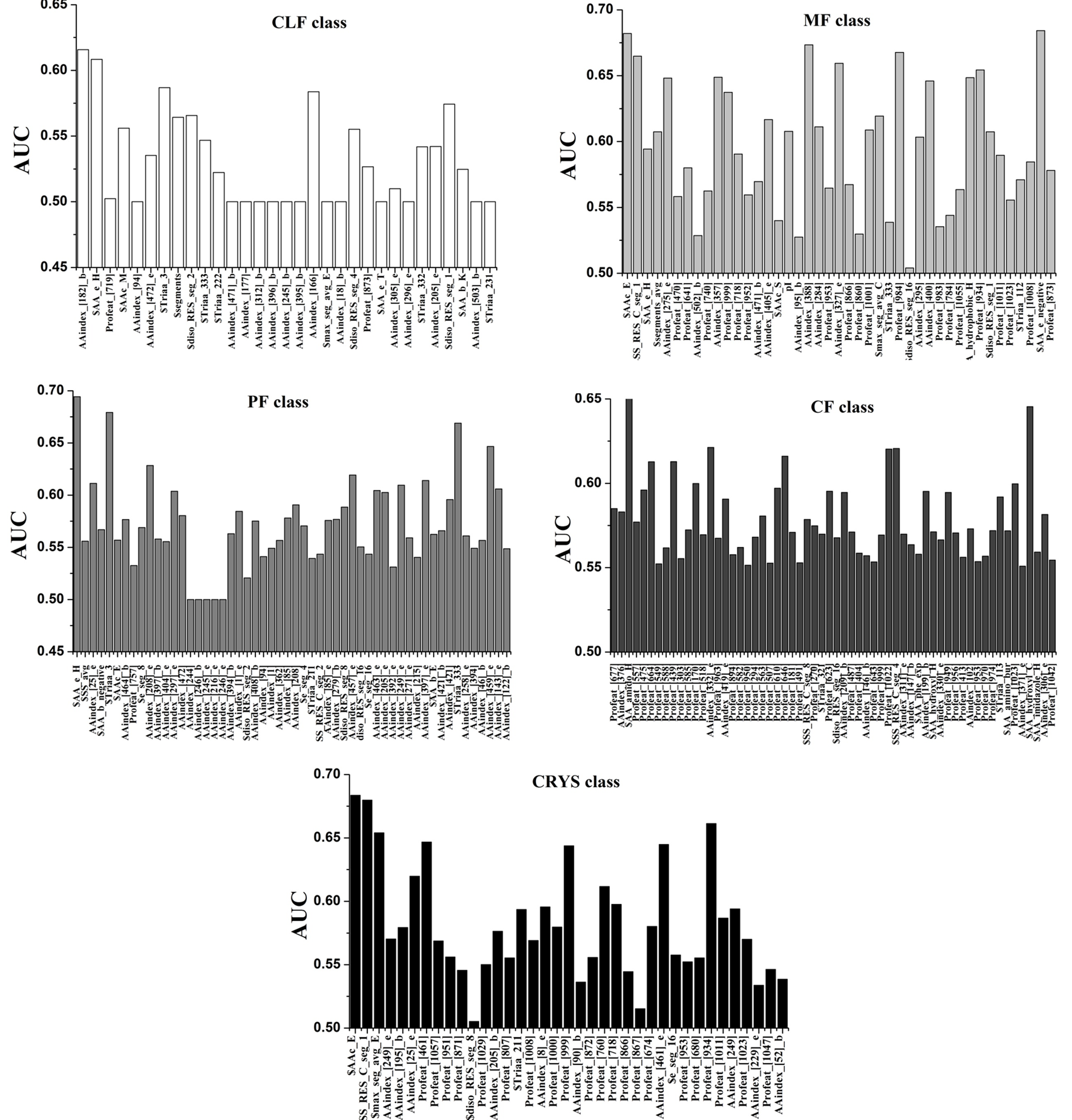

Supplement: Figure S1 — The importance and contribution of final selected features to the prediction performance of five classes, as evaluated by the AUC score using benchmark datasets. In particular, the optimal feature set of the CF class included 229 features and accordingly, we only displayed the features with AUC scores of larger than 0.55, in order to show the results more clearly. (TIF) [file pone.0105902.s001.tif]
