# Supplementary material for: PredPPCrys: Accurate Prediction of Sequence Cloning, Protein Production, Purification and Crystallization Propensity from Protein Sequences Using Multi-Step Heterogeneous Feature Fusion and Selection
Source: PLoS One. 2014 Aug 22;9(8):e105902. doi: 10.1371/journal.pone.0105902 (PMC4141844; doi:10.1371/journal.pone.0105902)
Supplement: Table S1 — Statistics of the 5-class assignment of protein targets marked with the corresponding statuses, including Sequence cloning failed (CF), Production of protein material failed (MF), Purification failed (PF), Crystallization failed (CF) and High-quality diffraction crystallization (CRYS). The data were extracted from PepcDB in this study. (DOCX) [file pone.0105902.s002.docx]

**Table S1.** **Statistics of the 5-class assignment of protein targets marked with the corresponding statuses, including Sequence cloning failed (CF), Production of protein material failed (MF), Purification failed (PF), Crystallization failed (CF) and High-quality diffraction crystallization (CRYS).** The data were extracted from PepcDB in this study.

| Labeled class | Sequence cloning failed | Production of protein material failed | Purification failed | Crystallization failed | Crystallizable |
| --- | --- | --- | --- | --- | --- |
| Final status | Selected | Cloned | Soluble | Crystallized | Crystal structure |
|  |  | Expressed | Purified | Diffraction | In PDB |
| Final target number | 11 351 | 8 945 | 2 794 | 258 | 5 383 |
| 5-class datasets |  |  |  |  |  |
| CLF classification | Negative | Positive | | | |
| MF classification | Negative |  | Positive | | |
| PF classification |  |  | Negative | Positive | |
| CF classification |  |  |  | Negative | Positive |
| CRYs classification | Negative |  |  |  | Positive |
