# Supplementary material for: PredPPCrys: Accurate Prediction of Sequence Cloning, Protein Production, Purification and Crystallization Propensity from Protein Sequences Using Multi-Step Heterogeneous Feature Fusion and Selection
Source: PLoS One. 2014 Aug 22;9(8):e105902. doi: 10.1371/journal.pone.0105902 (PMC4141844; doi:10.1371/journal.pone.0105902)
Supplement: Table S2 — A full list of all the considered sequence and sequence-derived features in this study. (DOCX) [file pone.0105902.s003.docx]

**Table S2. A full list of all the considered sequence and sequence-derived features in this study.**

| Feature class | Number | Feature name and its description |
| --- | --- | --- |
| AA type1 | 20 | Frequency of the 20 standard amino acid (AA) types |
| Buried/Exposed | 2 | Frequency of exposed/buried amino acids in the sequence |
| AA type1  & Buried/Exposed | 40 | Frequency of the 20 standard amino acid types in all the predicted buried/exposed residues in a given sequence |
| AAindex1 | 544 | The average value of physicochemical properties using the amino acids index (AAIndex1) over the whole sequence |
| AAindex1 & Buried/Exposed | 1088 | The average value of physicochemical properties using the amino acids index (AAIndex1) in all the predicted buried/exposed residues |
| AA type2 (hydrophobic/hydrophilic/neutral/position/negative) | 5 | Frequency of the hydrophobic (FIWLVMYCA)/hydrophilic (RKNDEP)/neutral (THGSQ)/position (KHR)/negative (DE) amino acids over the whole sequence |
| AA type2 & Buried/Exposed | 10 | Frequency of the hydrophobic/hydrophilic/neutral/position/negative amino acids in all the predicted buried/exposed residues |
| AA type3 (10 amino acid group) | 10 | Frequency of 10 amino acid groups in the sequence. We categorized 20 amino acids into 10 functional groups based on the presence of side chain chemical group such as phenyl (F/W/Y), carboxyl (D/E), imidazole (H), primary amine (K), guanidine (R), thiol (C), sulfur (M), amido (Q/N), hydroxyl (S/T) and nonpolar (A/G/I/L/V/P). |
| AA type3 & Buried/Exposed | 20 | Frequency of 10 amino acid groups with predicted buried or exposed residues |
| Tri-peptides & AA type2 (hydrophobic/hydrophilic/neutral) | 27 | Frequency of 27 tri-peptides composed of three amino acid grouping of hydrophobic, hydrophilic and neutral residues |
| Tri-peptides & charge | 4 | Frequency of tri-peptides with two or three charged amino acids |
| Sequence length | 1 | The total number of amino acid numbers in a given sequence |
| pI | 1 | The isoelectric point of protein |
| Side chain entropy & Buried/Exposed | 3 | The mean side chain entropy in the predicted buried/exposed residues |
| GRAVY & Buried/Exposed | 3 | The average hydrophobicity values (GRAVY) using the Kyte-Doolittle hydropathy parameters of the whole sequences or the predicted buried/exposed residues |
| PROFEAT | 1060 | Structural and physicochemical features of amino acid sequence by PROFEAT (excluding amino acids composition) |
| SS(C/H/E)_length | 3 | The average length of the predicted second structure (coil/helix/strand) segments divided by the sequence length |
| SS(C/H/E)_avg | 3 | The total number of predicted C/H/E segments divided by the sequence |
| SS(C/H/E)_max_length | 3 | The maximal length of the predicted coil/helix/strand segments divided by the sequence length |
| SS(C/H/E)_value | 3 | The average predicted probability of second structure over the whole sequence |
| SS(C/H/E)_length & Exposed/Buried | 6 | Number of the predicted secondary structure state length divided by the number of exposed/buried residues |
| SS(C/H/E)_length_seg | 15 | Number of the SS (C/H/E) residues in the secondary structures which are at least 1, 2, 4, 8 and 16 residues long, divided by the sequence length. |
| SS(C/H/E) & AA type3 | 30 | Frequency of 10 amino acid groups with predicted helix, strand and coil structures |
| SS(C/H/E) & AA type2 (hydrophobic/hydrophilic/neutral) | 9 | Frequency of hydrophobic, hydrophilic and neutral amino acids at helix, strand and coil |
| SS(C/H/E)_seg | 1 | Number of the predicted secondary structure segments, divided by sequence length |
| Disorder_value | 1 | The average value of the predicted disorder probabilities |
| Disorder_seg | 1 | Number of the predicted disorder segments |
| Disorder_length | 1 | The average length of the predicted disorder segments divided by the sequence length |
| Disorder_max_seg | 1 | The maximal length of the predicted disorder segment divided by the sequence length |
| Disorder_value/length & Exposed/Buried | 4 | The average value of the predicted disorder probability or disorder residue number for all the predicted exposed/buried residues |
| Disorder_length_seg | 5 | Number of the predicted disorder residues in the disorder segments which are at least 1, 2, 4, 8 and 16 residues long, divided by the sequence length. For segments with at least one residue, this feature represents content of the predicted disorder |
| Disorder & SS(C/H/E) | 1 | Frequency of disorder amino acids at helix, strand and coil in the sequence |
| Total |  | 2924 features for 5-class prediction |
