# Supplementary material for: PredPPCrys: Accurate Prediction of Sequence Cloning, Protein Production, Purification and Crystallization Propensity from Protein Sequences Using Multi-Step Heterogeneous Feature Fusion and Selection
Source: PLoS One. 2014 Aug 22;9(8):e105902. doi: 10.1371/journal.pone.0105902 (PMC4141844; doi:10.1371/journal.pone.0105902)
Supplement: Table S3 — List of the sequence-derived features used in other previous studies and our study. (DOCX) [file pone.0105902.s004.docx]

**Table S3.** List of the sequence-derived features used in other previous studies and our study.

| Featrue type | ParCrys | OBScore | CRYSTA P2 | XtalPred | SVMCR Ys^a^ | P_XS_ | PPCPred | PredPPCrys |  |  |  |  |
| --- | --- | --- | --- | --- | --- | --- | --- | --- | --- | --- | --- | --- |
|  |  |  |  |  |  |  |  | CLF | MF | PF | CF | CRYs |
| Dipeptide | 0 | 0 | 65 | 0 | 0 | 0 | 0 | 0 | 0 | 0 | 0 | 0 |
| Tripeptide | 0 | 0 | 19 | 0 | 1 | 0 | 0 | 5 | 2 | 3 | 2 | 1 |
| Isoelectric point | 1 | 1 | 1 | 1 | 0 | 0 | 0 | 0 | 1 | 0 | 0 | 0 |
| Hydrophobicity | 1 | 1 | 1 | 1 | 4 | 0 | 5 | 0 | 1 | 0 | 0 | 0 |
| Solvent accessibility | 0 | 0 | 0 | 0 | 0 | 0 | 3 | 16 | 8 | 35 | 6 | 10 |
| Secondary structure | 0 | 0 | 0 | 2 | 2 | 0 | 1 | 2 | 3 | 2 | 2 | 2 |
| Sidechain entropy | 0 | 0 | 0 | 0 | 0 | 1 | 0 | 0 | 0 | 0 | 0 | 0 |
| Disorder | 0 | 0 | 0 | 1 | 0 | 1 | 1 | 3 | 1 | 3 | 0 | 1 |
| AAindex | 0 | 0 | 0 | 0 | 0 | 0 | 8 | 15 | 11 | 38 | 8 | 10 |
| PROFEAT | 0 | 0 | 0 | 0 | 0 | 0 | 0 | 2 | 20 | 1 | 14 | 21 |
| AA group | 0 | 0 | 0 | 0 | 3 | 0 | 0 | 0 | 0 | 0 | 1 | 0 |
| Other | 0 | 0 | 0 | 6 | 1 | 0 | 0 | 1 | 1 | 0 | 0 | 0 |
| AA composition | Ser, Cys, Gly, Tyr, Met | - | Lys, Tyr | Cys, Met, Trp, Tyr, Phe | Ser | Phe, Gly | Cys, His, Ser | His, Met, Glu, Lys | Glu, His, Ser | Glu, His | His | Glu |

^a^ Top 10 features selected for SVMCRYs

For the purpose of prdiction of diffraction-quality crystals yield propensity from protein sequence, the selected feature sets of previous bioinformatics methods including ParCrys, OBScore, CRYSTAP2, XtalPred, SVMCRYs, Pxs and PPCPred, were classified and compared with our method. In addition, the selected feature sets for 5-class prediction in our study were also listed and summarized here.
