# Supplementary material for: PredPPCrys: Accurate Prediction of Sequence Cloning, Protein Production, Purification and Crystallization Propensity from Protein Sequences Using Multi-Step Heterogeneous Feature Fusion and Selection
Source: PLoS One. 2014 Aug 22;9(8):e105902. doi: 10.1371/journal.pone.0105902 (PMC4141844; doi:10.1371/journal.pone.0105902)
Supplement: Table S4 — Performance comparison of binary classification results of crystallizability on the independent test set of the XtalPred-RF method. (DOCX) [file pone.0105902.s005.docx]

Table S4. Performance comparison of binary classification results of crystallizability on the independent test set of the XtalPred-RF method.

|  | MCC | ACC (%) | SPEC (%) | SENS (%) |
| --- | --- | --- | --- | --- |
| XtalPred-RF^a^ | 0.470 | **74.0** | **78.0** | 69.0 |
| PredPPCrys^b^ | **0.478** | 73.9 | 74.1 | **73.7** |

^a^ Results obtained from Jahandideh *et al* [[1](#_ENREF_1)].

^b^ Performance of PredPPCrys using the final optimal features selected by multi-step heterogeneous feature selection.

**References**

1. Jahandideh S, Jaroszewski L, Godzik A (2014) Improving the chances of successful protein structure determination with a random forest classifier. Acta Crystallogr D 70: 627-635.
