# Supplementary material for: PredPPCrys: Accurate Prediction of Sequence Cloning, Protein Production, Purification and Crystallization Propensity from Protein Sequences Using Multi-Step Heterogeneous Feature Fusion and Selection
Source: PLoS One. 2014 Aug 22;9(8):e105902. doi: 10.1371/journal.pone.0105902 (PMC4141844; doi:10.1371/journal.pone.0105902)
Supplement: Table S5 — Summary results of target selection on presently non-crystallization proteins by PredPPCrys. (DOCX) [file pone.0105902.s006.docx]

**Table S5. Summary results of target selection on presently non-crystallization proteins by PredPPCrys.**

| Classification dataset | Pass all^a^ | Optimal | Suboptimal | Average | Difficult | Very difficulty |
| --- | --- | --- | --- | --- | --- | --- |
| CLF | 896 | 608 | 957 | 2541 | 5318 | 1926 |
| MF | 608 | 448 | 596 | 1444 | 3729 | 2728 |
| PF | 235 | 77 | 176 | 557 | 1741 | 235 |
| CF | 77 | 48 | 68 | 96 | 44 | 2 |
| Total | 1816 | 1181 | 1797 | 4638 | 10832 | 4891 |

We applied the best-performing PredPPCry predictors to rank and annotate those structural genomics targets (our extracted datasets) that have not successfully yielded diffraction-quality crystals yet. The statistics of prioritized structural genomics targets with different prediction cutoffs is shown in this table. The protein targets were annotated and classified as five difficulty levels, for ‘Optimal’ (with probability score ≥0.6), ‘Suboptimal’ (with 0.55≤probability score <0.6), ‘Average’ (with 0.45≤probability score <0.55), ‘Difficult’ (with 0.2≤probability score <0.45) and ‘Very difficult’ (with probability score <0.2), in an order from the easiest to the hardest.

^a^ Pass all denotes the number of protein targets were predicted to can be successful in sequence clone, protein material production, purification, crystallization and ultimately structural determination.
